# Near-complete photoluminescence retention and improved stability of InP quantum dots after silica embedding for their application to on-chip-packaged light-emitting diodes†

**DOI:** 10.1039/c8ra00119g

**Published:** 2018-03-12

**Authors:** Eun-Pyo Jang, Jung-Ho Jo, Min-Seok Kim, Suk-Young Yoon, Seung-Won Lim, Jiwan Kim, Heesun Yang

**Affiliations:** Department of Materials Science and Engineering, Hongik University Seoul 04066 Republic of Korea hyang@hongik.ac.kr; Department of Advanced Materials Engineering, Kyonggi University Suwon 16227 Republic of Korea jiwank@kyonggi.ac.kr

## Abstract

Silica is the most commonly used oxide encapsulant for passivating fluorescent quantum dots (QDs) against degradable conditions. Such a silica encapsulation has been conventionally implemented *via* a Stöber or reverse microemulsion process, mostly targeting CdSe-based QDs to date. However, both routes encounter a critical issue of considerable loss in photoluminescence (PL) quantum yield (QY) compared to pristine QDs after silica growth. In this work, we explore the embedment of multishelled InP/ZnSeS/ZnS QDs, whose stability is quite inferior to CdSe counterparts, in a silica matrix by means of a tetramethyl orthosilicate-based, waterless, catalyst-free synthesis. It is revealed that the original QY (80%) of QDs is nearly completely retained in the course of the present silica embedding reaction. The resulting QD–silica composites are then placed in degradable conditions such UV irradiation, high temperature/high humidity, and operation of an on-chip-packaged light-emitting diode (LED) to attest to the efficacy of silica passivation on QD stability. Particularly, the promising results with regard to device efficiency and stability of the on-chip-packaged QD-LED firmly suggest the effectiveness of the present silica embedding strategy in not only maximally retaining QY of QDs but effectively passivating QDs, paving the way for the realization of a highly efficient, robust QD-LED platform.

## Abstract 2

Silica embedding strategy enabling a nearly full PL retention of the original QY of InP QDs is proposed for the realization of a highly efficient, robust QD-LED platform.

## Graphical abstract

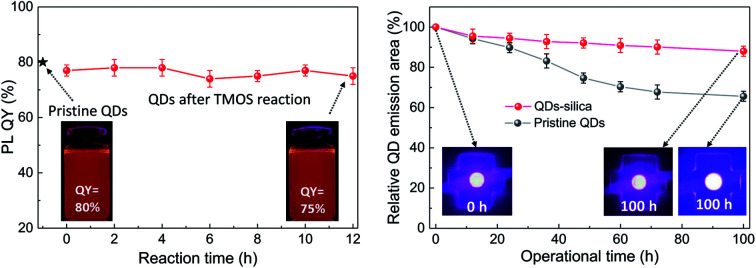

## Introduction

Based on substantial progress in the photoluminescent (PL) qualities of semiconductor quantum dots (QDs), which was achieved by the incessant development of colloidal synthetic methodology and sophisticated engineering of core/shell heterostructures, they have been highlighted as key materials for various optoelectronic devices including next-generation light-emitting diodes (LEDs), lasers, and luminescent solar concentrators.^1–3^ In particular, QDs have been already commercially applied to LCD backlight units as color-converting emitters in combination with a blue LED pumping source. In this commercialized version, QDs are dispersed in a large-area polymeric resin film and the resulting QD–resin composite is then sandwiched by two oxide-based gas barrier films in order to suppress the photodegradation of QDs from permeable oxygen and water vapor molecules. Moreover, for long-term reliability of this type of QD film assembly, often referred to as QD enhancement film (QDEF), blue LED is placed distantly from QDEF to avoid direct exposure of QDs to high temperature and high photon flux from LED at operation.^4^ However, QDEF is a costly design in that it acquires a large quantity of QDs and expensive gas barrier films. Therefore, a more cost-effective alternative such as direct QD integration onto an LED, also called as on-chip packaging, should be pursued. Together with huge advancement in PL attributes of QDs towards higher quantum yield (QY) and narrower emission bandwidth, their stability against degradable processings and conditions including repeated purification cycle, ligand exchange, prolonged UV irradiation, or thermal treatment has been steadily improved by the elaborate control on core/shell heterostructure mainly based on multi-shelling and/or thick- (or giant-) shelling strategies.^5–8^ However, in the case of on-chip-packaged QD-LEDs (herein, QD-LEDs stand for optically-pumped, color-converting devices, not electrically-driven ones), where more harsh environments of considerable heat plus intense photon flux emitting from LED chip are placed, even structurally strengthened QDs are not robust sufficiently, unexceptionally losing their initial fluorescent intensity over time of device operation.

The most viable strategy to protect QDs against degradable environments is to physically encase them with chemically stable oxide phases in the form of individual overcoating or collective embedding. The most widely used oxide for QD encapsulation is sol–gel-derived silica based on tetraethyl orthosilicate (TEOS) as a silane precursor, even though other non-silica oxide candidates such as In_2_O_3_ and ZnGa_2_O_4_ have been proposed to individually overcoat InP^9^ and Cu–In–S QDs,^10^ respectively. Silica phase can be produced as either overlayer^11–18^ or matrix,^19–24^ depending on its synthetic details of Stöber or reverse microemulsion (RM) process. However, both routes encounter a critical issue of considerable QY loss relative to pristine QDs after silica growth, since ligand exchange of QD for phase transfer and/or primer formation, hydrolyzed TEOS, and ammonia catalyst likely lead to the chemical devastation of QD surface.^11,14,16,18,19^ Therefore, a few notable strategies to mitigate such a silica processing-accompanying side effect have been developed. Jun *et al.* adopted the combination of 6-mercaptohexanol as ligand exchange reagent and propylamine as a mild base catalyst for an effort to minimize the oxidative damage of QD surface in the course of silica processing, enabling the formation of a highly luminescent and photostable CdSe/CdS/ZnS QD–silica monolith.^25^ Murase group suggested that the silanization (or ligand exchange) of hydrophobic Cd-based QDs with partially hydrolyzed TEOS species was a crucial pre-treatment step prior to silica growth reaction in not only retaining the original QY of QDs maximally but ensuring the homogenous formation of silica shell.^14^ Meanwhile, as a simple, non-chemical approach to the formation of QD–silica composites, pre-prepared micron-sized mesoporous silica particles were employed as physical templates, wherein Cu–In–S or perovskite CsPbBr_3_ QDs could be infiltrated into the pores by means of swelling and solvent evaporation method.^26,27^

Although QY and emission bandwidth of non-toxic (*i.e.*, Cd-free) InP QDs are fast approaching those of CdSe-based counterparts, the former QDs are quite inferior in stability against degradable conditions to the latter ones. On that account, InP QDs are more susceptible to the silica reaction condition and thus may suffer from a more PL quenching after silica growth compared to CdSe-based ones. For instance, the original QY (30–50%) of red InP/ZnS QDs became severely deteriorated to 15% after RM-processed silica overcoating.^12^ Therefore, a special silica processing for such delicate InP QDs to enable the maximal retention of original QY is demanded, but work on the formation of InP QD–silica composites has been rarely reported to date. Very recently, a simple, novel silica encapsulation for perovskite CH_3_NH_3_PbBr_3_ QDs, which are highly vulnerable to contact with various polar solvents due to their strong ionic nature, has been developed by a waterless, catalyst-free synthesis, where the trace amount of water present in toluene solvent was used to hydrolyze tetramethyl orthosilicate (TMOS) at room temperature (25 °C) in a sealed reactor.^28^ On the basis of the above strategy for the formation of QDs–silica, in this work, we explore the embedment of multishelled InP/ZnSeS/ZnS QDs in silica matrix with a synthetic modification. Instead of the sealed system aforementioned, we adopt the open system, where silica reaction is directly exposed to a relative humidity (RH) of 70% at 30 °C. It is revealed that under this condition the original QY of QDs is well preserved throughout silica reaction. To attest to the efficacy of silica encapsulation on QD stability the resulting InP QD–silica composites are then applied as color-converters with a blue LED in on-chip-packaging, showing 88% retention of the initial QD emission after a long-term operation of 100 h at a driving current of 60 mA.

## Experimental

### Synthesis of multishelled InP/ZnSeS/ZnS QDs

Our multishelled InP/ZnSeS/ZnS QDs were prepared by following our previous synthetic protocol^29^ with a slight modification. For a typical preparation of red-emitting InP core QDs, 0.45 mmol of indium chloride (InCl_3_), 2.2 mmol of zinc chloride (ZnCl_2_) and 6 ml of oleylamine (OLA) were placed in a three-necked flask, and then this mixture was degassed at 120 °C for 60 min and further heated to 180 °C under nitrogen flow. At that temperature, 0.35 ml of tris(dimethylamino)phosphine (P(N(CH_3_)_2_)_3_, P(DMA)_3_) was swiftly injected and the reaction was maintained for 25 min. Consecutive growth of composition-gradient ZnSeS intermediate shell proceeded by the repeated alternate injections of anionic and cationic shell precursors by the following manners. The Se stock solution, prepared by dissolving 0.12 mmol of selenium (Se) in 1 ml of trioctylphosphine (TOP), was introduced and reacted at 200 °C for 30 min. And the Zn stock solution, prepared by dissolving 1.58 mmol of zinc stearate in 4 ml 1-octadecene (ODE), was injected, followed by the reaction at 220 °C for 30 min. Then, the Se–S stock solution (0.06 mmol of Se and 2 mmol of sulfur (S) dissolved in 1.6 ml of TOP) was injected and reacted at 240 °C for 30 min and then the above Zn stock solution was introduced, followed by the reaction at 260 °C for 30 min. For the last ZnSeS intermediate shelling, another S-richer Se–S stock solution (0.02 mmol of Se and 4 mmol of S dissolved in 2 ml of TOP) was added and reacted 280 °C for 30 min, followed by the injection of the same Zn stock solution and the reaction at 300 °C for 60 min. For the deposition of ZnS outer shell, 5 ml of 1-dodecanethiol was slowly introduced and reacted at 200 °C for 60 min, and then another Zn stock solution, prepared by dissolving 3 mmol of Zn acetate in 3 ml of oleic acid, was injected, followed by the reaction at 190 °C for 120 min. After cooling the reaction, as-synthesized multishelled InP/ZnSeS/ZnS QDs were placed in repeated purification cycles by a precipitation/redispersion with an ethanol/hexane combination using centrifugation (8000 rpm, 15 min for each) and finally redispersed in hexane or toluene.

### Preparation of QD–silica composites

To 2 ml of toluene dispersion of InP/ZnSeS/ZnS QDs having an optical density (OD) of 2.0 adjusted at 574 nm was added 18 ml of toluene, resulting in 20 ml of dilute QD dispersion with an OD with 0.2 with the same wavelength. 1 ml of TMOS was added to the above dispersion. Then, this mixture was placed and stirred in a thermohygrostat (TH-PE-100, JEIO Tech., Korea) set at a temperature of 30 °C and a RH of 70%. This silica embedding reaction proceeded typically for 20–24 h, where the flocculation of QDs–embedded silica was observed in the solution. Such flocculated particles were precipitated by centrifugation (8000 rpm, 10 min) and washed twice with an excess of acetone.

### Stability tests and QD-LED fabrication

Both pristine QDs and QDs–silica became powdered by completely drying them in a vacuum oven at 60 °C for the following QD stability tests and QD-LED fabrication. Two comparative powdered QDs were closely packed into a stainless sample holder (diameter: 7 mm, depth: 0.5 mm) and then were identically exposed to degradable conditions of UV irradiation and 85 °C/85% RH for certain periods of time using a 365 nm-multi-band UV lamp (225 mW cm^−2^) and a thermohygrostat, respectively.

For the fabrication of QD-LEDs, *ca.* 6 mg of pristine QDs and *ca.* 18 mg of QDs–silica were individually mixed first with 0.3 g of thermally curable epoxy resin (YD-128, Kukdo Chem., Korea). To these QD–resin mixtures was added 0.3 g of a hardener (KFH-271, Kukdo Chem., Korea). The resulting pastes were dispensed into a 5 mm × 5 mm-sized, surface-mounted typed, blue InGaN LED (*λ* = 455 nm, Dongbu LED Inc.) mold and then hardened by a sequential thermal curing process of 90 °C for 1 h and 120 °C for 30 min (Fig. S1†).

### Characterization

Absorption spectrum of QDs was recorded by using UV-visible absorption spectroscopy (Shimadzu, UV-2450). PL spectra of QDs and QDs–silica in the forms of dispersion and powder were collected with a 500 W Xe lamp-equipped spectrophotometer (PSI Co. Ltd., Darsa Pro-5200). Absolute PL QYs of QDs in dispersion were assessed at an excitation wavelength of 450 nm in an integrating sphere by using an absolute PL QY measurement system (C9920-02, Hamamatsu). Relative QYs were also measured by calculating the integrated emission of QD sample in dispersion relative to that of rhodamine 6G (PL QY of 95%) in ethanol at an identical OD, resulting in the same values as in absolute ones. PL lifetimes were measured by employing the time-correlated single-photon counting (TCSPC) method on a FS5 spectrophotometer (FS5, Edinburgh Instruments) equipped with an EPL-375 nm picosecond pulsed diode laser. Transmission electron microscopic (TEM) images of QDs and QD–silica composites were taken by a JEM-2100F (JEOL Ltd.) operating at 200 kV. Electroluminescent (EL) data of QD-LEDs fabricated such as EL spectrum and luminous efficacy (LE) were acquired with a diode array rapid analyzer system (PSI Co. Ltd) in an integrating sphere.

## Results and discussion

**Fig. 1:**
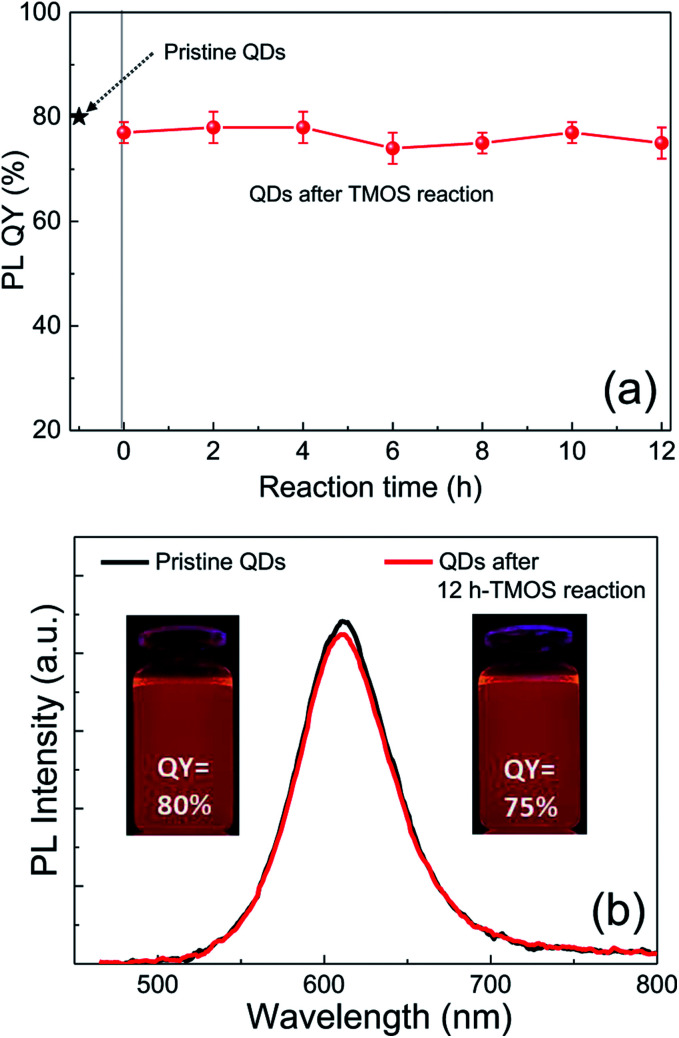
(a) Temporal change of PL QY of InP/ZnSeS/ZnS QDs after TMOS addition. The original QY (80%) of pristine QDs was marked with a star. The error bars represent five time-repeated measurements. (b) Comparison of PL spectra and UV-irradiated fluorescent images of pristine and 12 h-TMOS-reacted QD dispersions (in toluene). The excitation wavelength for PL was 450 nm.

**Fig. 2:**
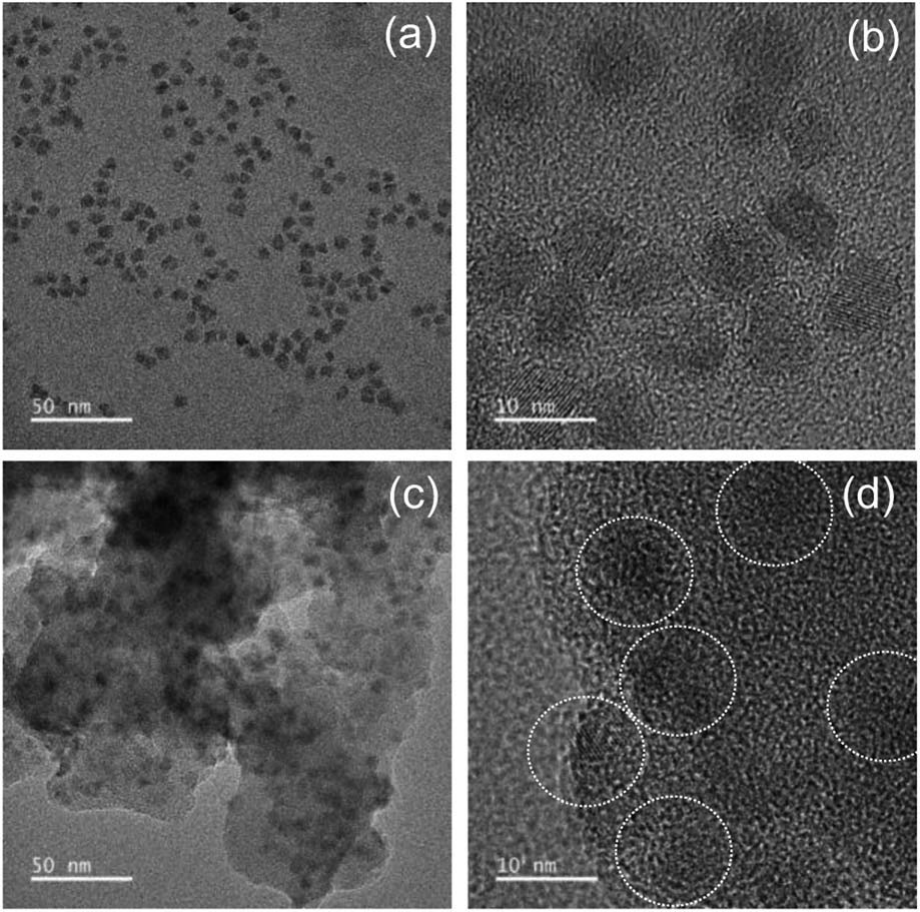
(a and c) Lower- and (b and d) high-magnification TEM images of pristine QDs (a and b) and QDs–silica (c and d).

Although silica in the form of overlayer or matrix is effective in passivating QDs from oxidative environments, most of QDs experience an inevitable PL quenching during silica reaction, which proceeds mostly by the conventional Stöber or RM protocol. Degree of PL loss appears rather sensitive to the heterostructure of QDs^18^ and silica processing detail.^14,16^ In this regard, we first investigated the effect of the present silica processing on QY of pristine QDs by monitoring temporal variation in QY over reaction time. Our pristine red InP/ZnSeS/ZnS QDs, consisting of an intermediate shell of composition-gradient (*i.e.*, ZnS-richer phase outward) ZnSeS and an outer shell of ZnS, with a peak wavelength of 611 nm possess the original QY of 80%. Their absorption spectrum is also presented in Fig. S2† for reference. Only a slight change in QY from 80 to 77% was observed upon introducing 1 ml of TMOS into 20 ml of QD toluene dispersion with an OD of 0.2 at 574 nm, and the overall QY values were well retained in the range of 75–78% even after extended periods of reaction (Fig. 1a). As compared in Fig. 1b, PL spectrum of the QD solution after 12 h-TMOS reaction stayed nearly unchanged compared to that of the pristine QD solution. To the best of our knowledge, this is the unprecedented result demonstrating the formation of InP QD–silica composites with the original QY nearly intact. Note that the present silica reaction was performed in a high RH of 70% to facilitate the hydrolysis of TMOS, unlike in silica embedding of perovskite QDs, where a very small amount of water content contained in toluene was used for its hydrolysis (*i.e.*, the sealed system).^28^ As a preliminary experiment, silica reaction was attempted in the sealed system, but there was no sign for the generation of silica phase regardless of the amount of TMOS and reaction time. Additionally, TEOS was used instead of TMOS with other reaction conditions unchanged. Intriguingly, injection of TEOS into QD toluene dispersion led to an instantaneous QY drop down to 52%, after which this value was largely maintained within the range of 50–55% (Fig. S3†). Such a considerable QY drop during TEOS reaction should be correlated to its substantially slow hydrolysis rate compared to TMOS.^28^ TEOS molecules, which stay unhydrolyzed for a long period of time, likely devastate the surface of QDs presumably *via* removal of organic surface ligands throughout the reaction prior to the formation of silica phase. On the other hand, TMOS molecules that can be rapidly hydrolyzed can give rise to a faster silica formation, preventing the deterioration of QD surface and thus well retaining QY. As shown in transmission electron microscopic images (Fig. 2a and b), our InP/ZnSeS/ZnS QDs exhibit a distinctively large size of 7 nm compared to other single-^30,31^ and multishelled InP QDs^5,32^ with typical diameters <5.5 nm. This implies that the present ZnSeS intermediate-shelling strategy in a composition-gradient fashion should lead to the effective relief of substantial interfacial strain between InP core and ZnS outer shell with a large lattice mismatch of 7.7%, thus allowing for a thick shell growth. Fig. 2c and d present low- and high-magnification TEM images, respectively, of QD–silica composites, where individual QDs were well buried in silica matrix. QDs in the matrix were quite evenly distributed without notable QD agglomerates that can cause a nontrivial PL quenching *via* Förster resonant energy transfer (FRET). These InP/ZnSeS/ZnS QD–silica composites were completely dried into a powder form (Fig. S4†) for the following stability tests in degradable conditions.

**Fig. 3:**
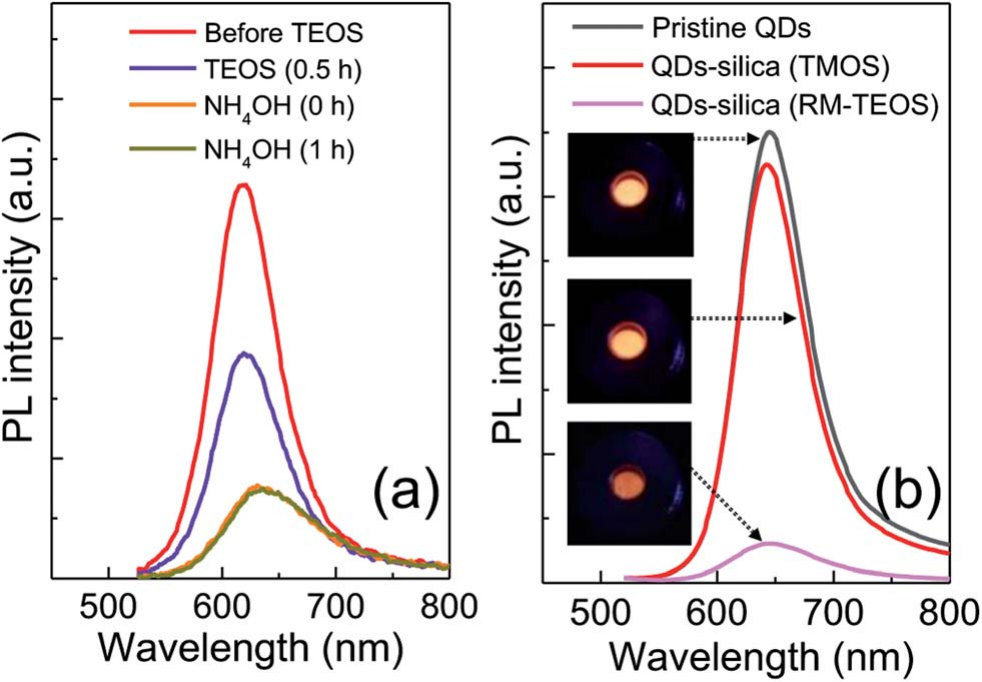
(a) Temporal change in PL intensity of InP/ZnSeS/ZnS QDs in the course of RM-based silica reaction consisting of TEOS and subsequent NH_4_OH addition. (b) Comparison of PL intensity of three powder samples of pristine QDs together with two QDs–silica composites from TMOS-based, catalyst-free *versus* TEOS-based RM methods. The excitation wavelength for all PLs was 450 nm.

To prove the superiority of the present silica embedding strategy in preserving the original QY, an archetypal RM method, where the presence of ammonia catalyst and water is necessary to produce silica phase, was applied to encapsulate InP/ZnSeS/ZnS QDs by adopting the protocols in literature (see ESI for details†).^12,17^ Analogous to the detrimental effect of TEOS on QY of QD toluene dispersion, just addition of TEOS into QD cyclohexane dispersion gave rise to a marked PL loss. And the consecutive introduction of NH_4_OH for hydrolysis and condensation further abated PL intensity of the QD dispersion (Fig. 3a). Such a huge drop in PL intensity of InP QDs, which is also consistent with the result (30–50% → 15% in QY) in literature,^12^ is likely unavoidable, even though some synthetic parameters such as NH_4_OH amount are controlled. For an intent to mitigate PL loss in the course of RM-based silica reaction, two different amounts of NH_4_OH (28 wt%) were tested. However, the variation of NH_4_OH amount was not helpful in minimizing PL loss, although it affected the morphology of QD–silica composites (Fig. S5†). Specifically, with increasing NH_4_OH amount the phase fraction of silica in QD–silica composites increased accordingly without changing QD size. Fig. 3b show PL spectral comparison of three samples in a powder form of pristine InP/ZnSeS/ZnS QDs together with two QD–silica composites obtained through TMOS-based, catalyst-free *versus* TEOS-based RM methods. While the former QD–silica composites were comparable in PL intensity to pristine QDs, the latter ones exhibited a considerably low PL intensity. This clearly indicates our TMOS-based, catalyst-free approach is highly advantageous in maximally retaining the original QY of QDs by effective preventing the devastation of QD surface accompanied by the conventional silica reaction.

To demonstrate the efficacy of silica embedding on QD stability, two comparative powder samples of pristine QDs *versus* QDs–silica were first exposed to UV irradiation in air atmosphere. After the elapse time of 15 h QDs–silica maintained 80% of the initial PL intensity, while a more marked reduction (42%) of PL was observed from pristine QDs (Fig. S6†). Followingly, the same samples were placed in a thermohygrostat set at 85 °C and 85% RH for an extended period of time up to 144 h, consistently showing a much higher PL retention of QDs–silica (30% reduction) over pristine QDs (53% reduction) (Fig. S7†). These comparative PL retention results of InP/ZnSeS/ZnS QDs without *versus* with silica encapsulation are by and large in line with those obtained from the earlier CdSe-based QDs.^15,16,18^ That is, although silica well serves as a physical barrier to protect QDs against degradable environments and thus impede the devastation of QD surface, a full retention in PL after silica encapsulation in the form of embedding or overcoating is unlikely. This is presumably because sol–gel-derived amorphous silica consists of a porous network structure (with a porosity of 10–15%),^23^ where oxygen and water vapor molecules become accessible to QD surface, followed by its oxidation and/or corrosion.

**Fig. 4:**
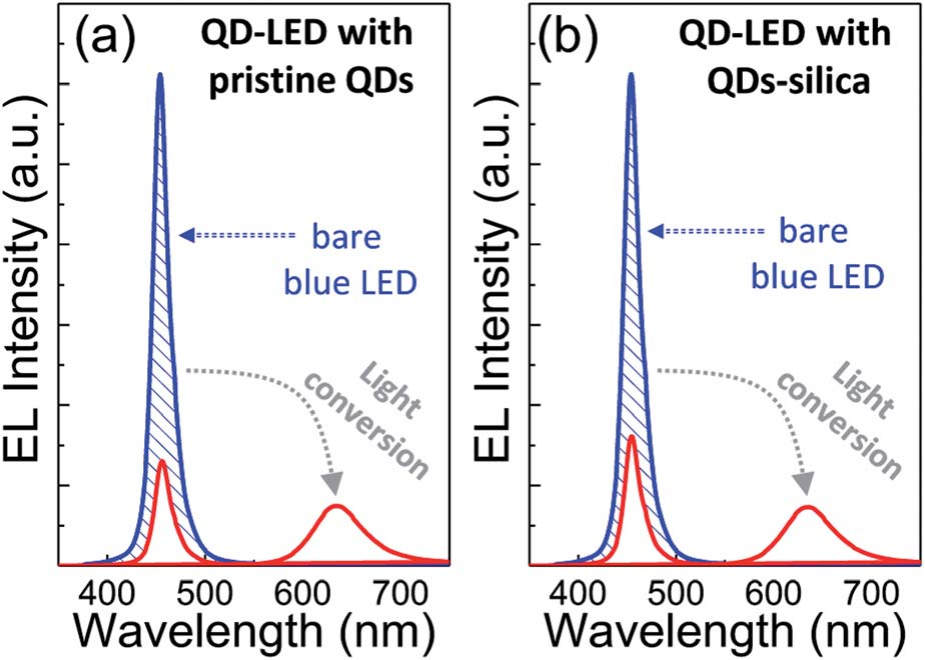
Comparison of EL spectra of on-chip-packaged QD-LEDs with pristine QDs and QDs–silica collected at an input current of 60 mA. An EL spectrum of 60 mA-driven bare blue LED was also collected for the calculation of blue-to-red light conversion efficiency.

**Fig. 5:**
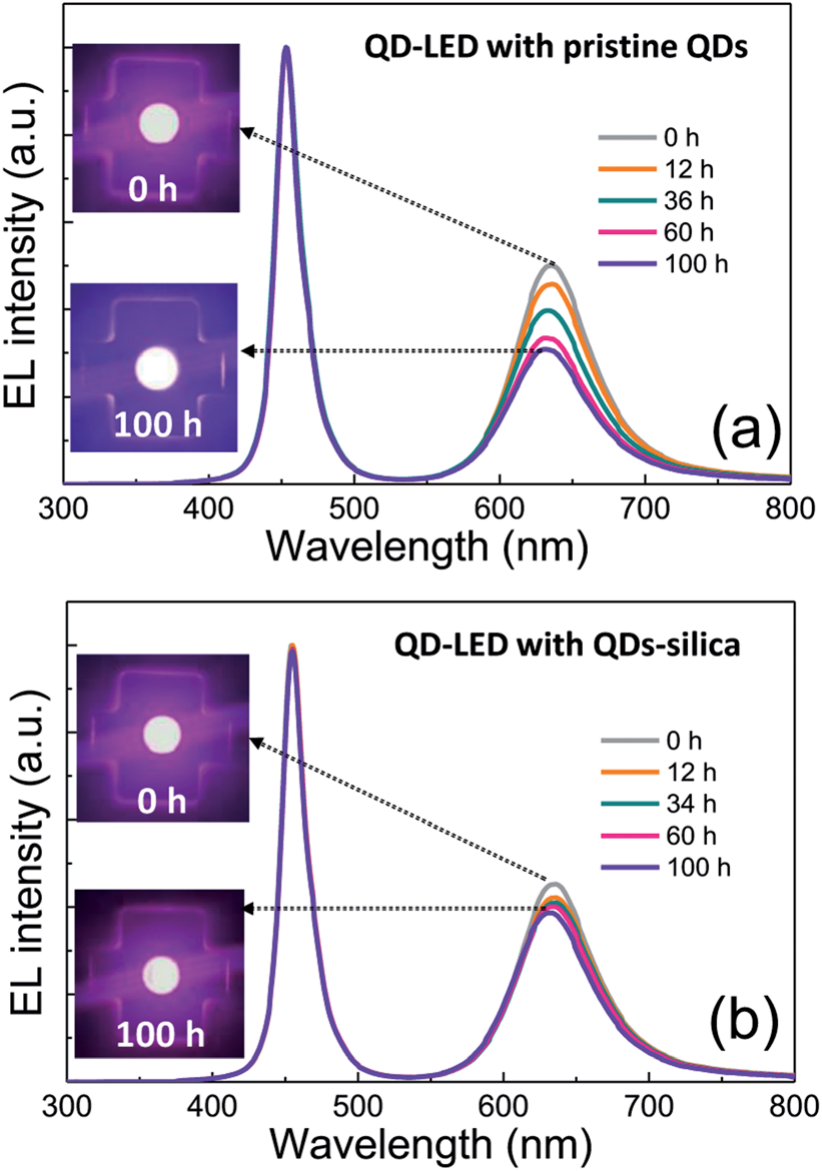
Operational time-dependent EL spectral evolutions of QD-LEDs with (a) pristine QDs and (b) QDs–silica at a driving current of 60 mA. The EL images operating in the initial period *versus* after 100 h-driving are compared in the left insets.

**Fig. 6:**
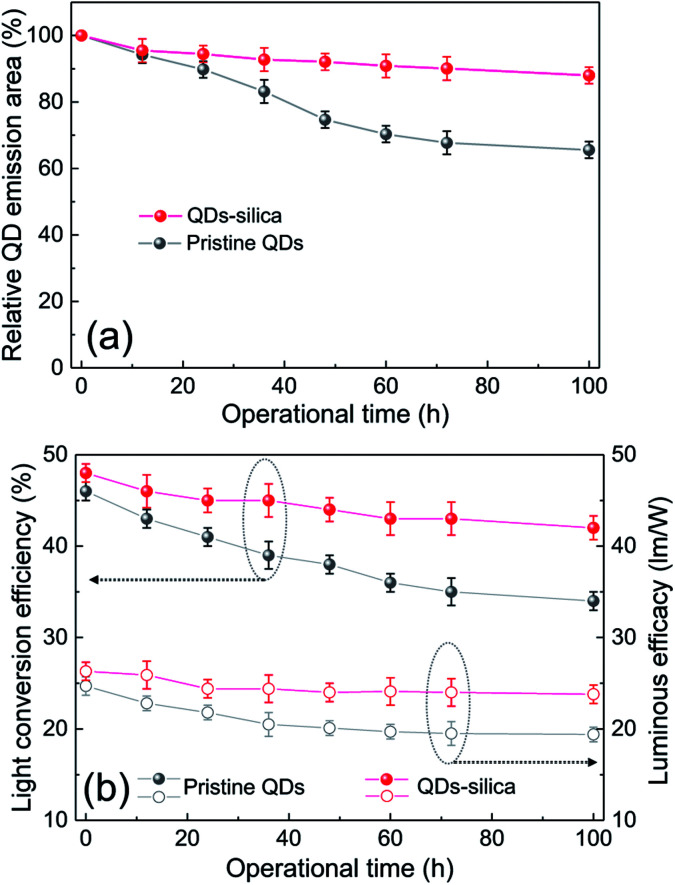
Changes in (a) relative QD emission area and (b) light conversion efficiency−luminous efficacy of QD-LEDs with pristine QDs and QDs–silica as a function of operational time at a driving current of 60 mA.

Two powder samples of pristine QDs and QDs–silica were individually blended with an epoxy resin and on-chip-packaged in a blue LED chip, and their EL spectra collected at a relatively high input current of 60 mA were compared in Fig. 4. Here, the light conversion efficiency (LCE) of QD-LED is assessed as the spectral integration ratio of the converted QD emission to the blue LED emission spent for that conversion. The QD-LEDs with pristine QDs and QDs–silica exhibited LCE values of 46 and 48%, respectively. Such similar LCEs imply that QDs–silica were comparable in PL QY to pristine QDs, again proving that our silica reaction strategy was highly beneficial in nearly retaining the original QY of pristine QDs. As inferred from Fig. 2, the average inter-QD spacing in on-chip-packaged devices would be more distant for QD–silica powder relative to pristine QD one, thus expecting that quenching of QD emission by means of FRET can be mitigated for the former compared to the latter.^23,33^ This speculation may be also supported by PL decay measurements showing average lifetime (*τ*_avg_) values 48 and 51 ns for pristine QDs and QDs–silica samples, respectively (Fig. S8†). In general, on-chip-packaged QD-LEDs suffer from the light scattering resulting from either aggregation of QDs in polymeric resin or mismatch in refractive index (RI) of QDs with resin, reducing the photon flux outwards.^17,34^ Particularly, such a gap in RI may be alleviated by forming silica-based QD composites and producing an intermediate RI value between those of QD and resin, depending on their volume fraction.^15,17^ Hence, these two factors, *i.e.*, reductions of FRET and light scattering, are likely jointly responsible for a slightly better LCE of QDs–silica- based device compared to pristine QDs-based one. The QD-LEDs with different loading amounts (*i.e.*, *ca.* 13 and 25 mg) of QDs–silica powders were additionally fabricated, showing decreases in blue-to-QD spectral ratio and LCE with increasing QDs–silica loading (Fig. S9†). Such an LCE reduction is attributable to a more active light reabsorption from more concentrated QDs–silica powders packaged in a LED mold. Two comparative QD-LEDs presented in Fig. 4 were subsequently subjected to the continuous operation at 60 mA for a prolonged period of time up to 100 h. As compared in temporal EL spectral evolutions (Fig. 5), only slight reductions of QD emission intensity was observed from the device with QDs–silica, whereas one with pristine QDs exhibited more progressive degrees of QD emission quenching. As a result, compared to the former device, a more marked EL color change after 100 h-operation was recognizable from the latter one (insets of Fig. 5a and b). Changes of relative QD emission area were also evaluated with operational time, showing 65 and 88% retention of their initial values after 100 h-operation for QD-LEDs without *versus* with silica embedding, respectively (Fig. 6a). Note that these operational stability tests were repeated by taking the same four devices of each QD-LED and the resulting marginal variations were presented in the error bars. These comparative tests on long-term operational device stability, which are consistent with the earlier ones on QD stability under UV irradiation and 85 °C/85% RH, manifest the efficacy of silica barrier in protecting QDs against the present degradable LED operational environments, *i.e.*, high blue photon flux and chip temperature of *ca.* 50 °C (accompanied by 60 mA-LED driving current) in air ambient. Atmospheric gaseous species are allowed to be readily accessible to QDs–silica due to a polymeric loose structure of epoxy encapsulant (used for QD packaging) and further permeate into QDs *via* porous channels of silica (aforementioned), incurring the photochemical degradation of QDs under a high photon flux from a blue LED chip. On that account, the photodegradation of QDs even encased in silica matrix will inevitably occur, but its rate should be substantially slowed down, considering only 12% loss of the initial QD emission intensity after 100 h-operation. According to the results of Fig. 6a, temporal variations in LCE were calculated, showing 46 → 34% and 48 → 42% after 100 h-operation for QD-LEDs without and with silica embedding, respectively (Fig. 6b). Initial (or 0 h-driving) LEs were higher for the device with QDs–silica (26.3 lm W^−1^) compared to that with pristine QDs (24.7 lm W^−1^) (Fig. 6b). Even though there is a marginal mismatch in blue-to-red EL spectral ratio of those two devices (Fig. 5), this increase in LE is a direct consequence of an enhanced LCE concomitant with the formation of QDs–silica aforementioned. Changes of LE with operational time also followed the same trends as those of relative QD emission intensity or LCE, showing temporal LE reductions of 24.7 → 19.4 lm W^−1^ and 26.3 → 23.8 lm W^−1^ from 0 h- to 100 h-driving for devices with pristine QDs and QD–silica, respectively (Fig. 6b). The above promising results on device efficiency and stability are entirely attributable to the effectiveness of the present silica embedding strategy in not only maximally retaining QY of QDs but effectively passivating QDs, which thus convincingly offers a practical means to realize the fabrication of a highly efficient, robust QD-LED system.

## Conclusions

First, we synthesized highly efficient red InP QDs with a composition-gradient ZnSeS intermediate shell and a ZnS outer shell. Silica embedding reaction proceeded by introducing TMOS only to QD toluene dispersion without additional catalyst and water and subsequently exposing it directly to 70% RH at 30 °C. It turned out that under the present silica reaction condition the original QY (80%) of QDs was well preserved, showing the overall QY values of 75–78% even after extended periods of reaction. Such a nearly complete PL retention, which has been not achievable for InP QDs and even CdSe ones through archetypal Stöber and RM processes, can be attributed to a chemically mild environment of the present TMOS-based, catalyst-free silica reaction, effectively preventing the devastation of QD surface. Two comparative powder samples of pristine QDs *versus* QD–silica composites were then exposed to UV irradiation and 85 °C/85% RH for prolonged periods of time, commonly exhibiting much higher PL stability behaviors from the latter relative to the former as a consequence of the effective physical protection of QDs with silica barrier against degradable environments. Two types of on-chip-packaged QD-LEDs fabricated with pristine QDs and QDs–silica displayed comparable blue-to-red LCE values of 46 and 48%, respectively, again supporting a nearly full retention of the original QY after silica embedding. The efficacy of silica embedding on the improvement of device stability was testified by a continuous operation at 60 mA up to 100 h. The QD-LEDs with pristine QDs *versus* QDs–silica exhibited markedly different degrees in the retention of QD emission and the reduction of LE, *i.e.*, 65 and 88% (in relative QD emission area) and 24.7 → 19.4 lm W^−1^ and 26.3 → 23.8 lm W^−1^, respectively, after 100 h-operation.

## Conflicts of interest

There are no conflicts to declare.

## Supplementary Material

RA-008-C8RA00119G-s001
