# Supplementary material for: Near-complete photoluminescence retention and improved stability of InP quantum dots after silica embedding for their application to on-chip-packaged light-emitting diodes
Source: RSC Adv. 2018 Mar 12;8(18):10057–63. doi: 10.1039/c8ra00119g (PMC9078749; doi:10.1039/c8ra00119g)
Supplement: RA-008-C8RA00119G-s001 [file RA-008-C8RA00119G-s001.pdf]

Electronic Supplementary Material (ESI) for RSC Advances.
This journal is © The Royal Society of Chemistry 2018

Supporting Information

Near-complete photoluminescence retention and improved stability of InP quantum dots after silica embedding for their application to on-chip-packaged light-emitting diode

Eun-Pyo Jang,[a‡] Jung-Ho Jo,[a‡] Min-Seok Kim,[a] Suk-Young Yoon,[a] Seung-Won Lim,[a] Jiwan Kim*[b] and Heesun Yang*[a]

[a] Department of Materials Science and Engineering, Hongik University, Seoul 04066, Republic of Korea

[b] Department of Advanced Materials Engineering, Kyonggi University, Suwon 16227, Republic of Korea

Preparation of QD-silica composites via reverse microemulsion method

In a typical preparation of QD-silica composites by reverse microemulsion method, 1.7 ml of IGEPAL 520 was first dissolved in 10 ml of cyclohexane. To this solution was added 0.1 ml of toluene dispersion of InP/ZnSeS/ZnS QDs having an optical density of 2.0 adjusted at 574 nm. Subsequently, 80 μl of tetraethyl orthosilicate (TEOS) was injected into the above mixture, followed by stirring for 30 min. Then, 0.04 or 0.3 ml of ammonia (28 wt%) was added to initiate the hydrolysis of TEOS, and the reaction proceeded for 24 h. The resulting QD-silica composites were collected by centrifugation (8000 rpm, 10 min) and washed twice with an excess of acetone.

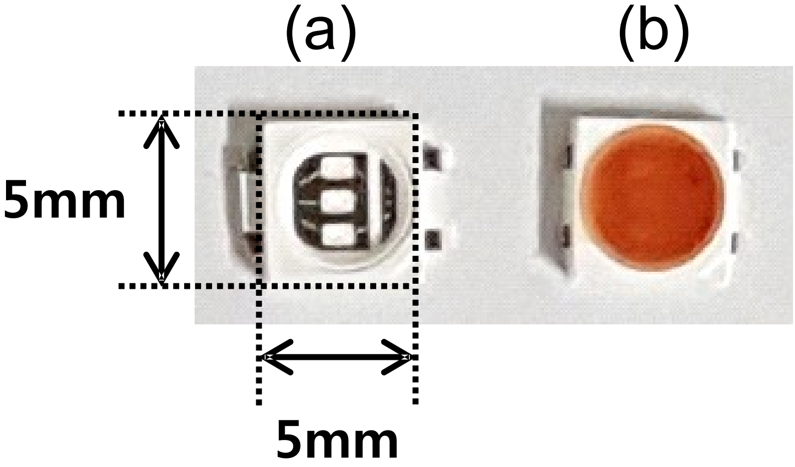

Fig. S1 Photos of (a) bare blue LED and (b) on-chip-packaged QD-LED.

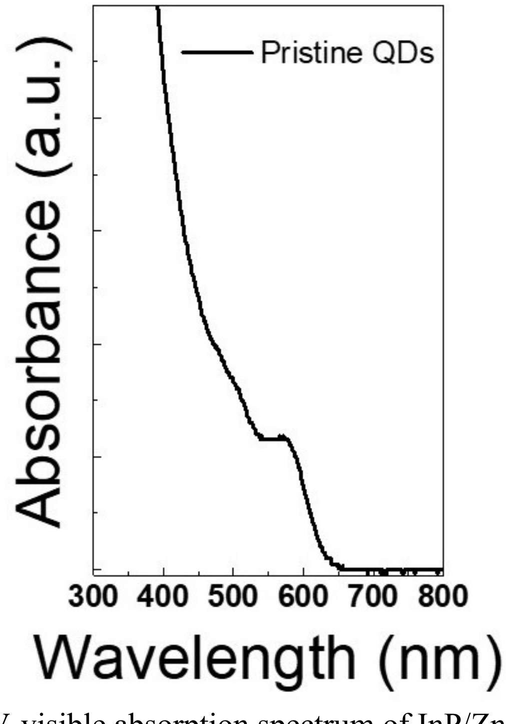

Fig. S2 UV-visible absorption spectrum of InP/ZnSeS/ZnS QDs.

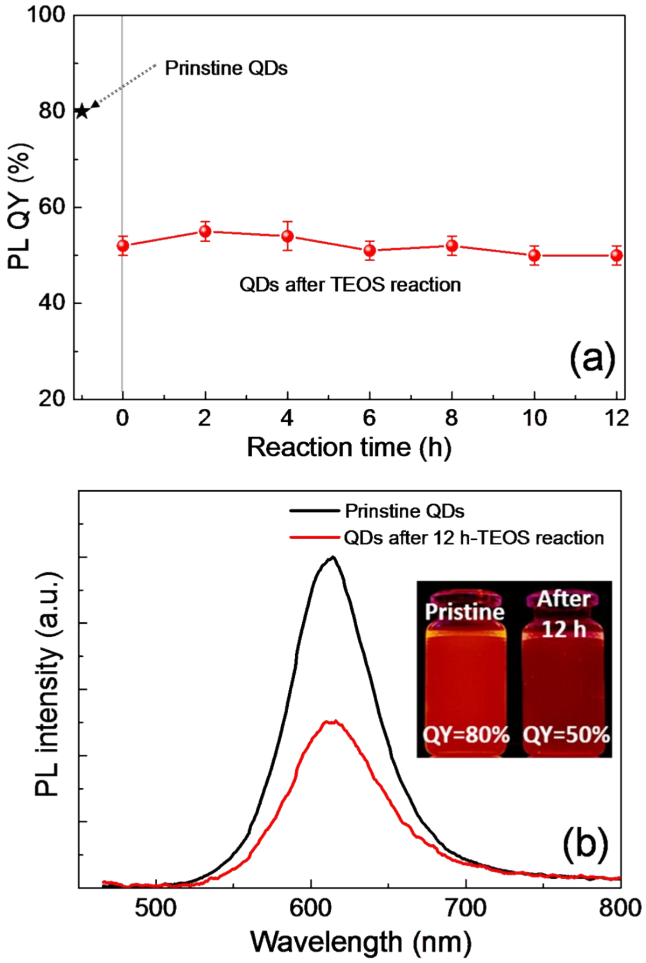

Fig. S3 (a) Temporal change of PL QY of InP/ZnSeS/ZnS QDs after TEOS addition. The original QY (80%) of pristine QDs was marked with a star. The error bars represent three time-repeated measurements. (b) Comparison of PL spectra and UV-irradiated fluorescent images of pristine and 12 h-TEOS-reacted QD dispersions (in toluene). The excitation wavelength for PL was 450 nm.

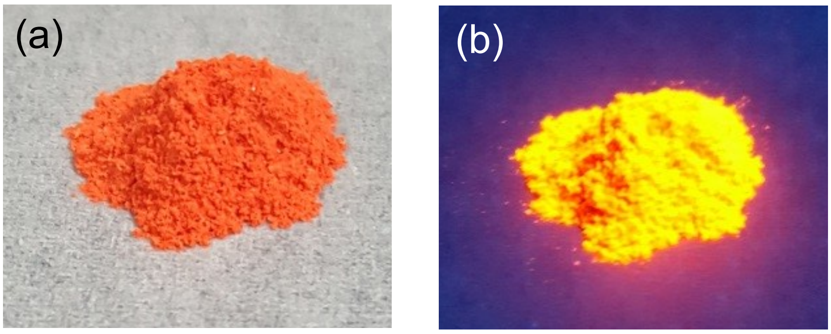

Fig. S4 Powdered sample of InP/ZnSeS/ZnS QDs-silica composites under (a) room light and (b) a hand-held UV lamp.

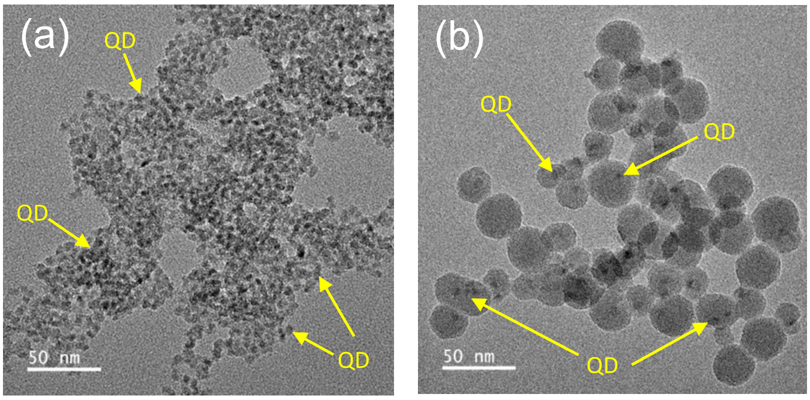

Fig. S5 TEM images of RM method-derived QDs-silica composites prepared with (a) 0.04 and (b) 0.3 ml of ammonia (28 wt%).

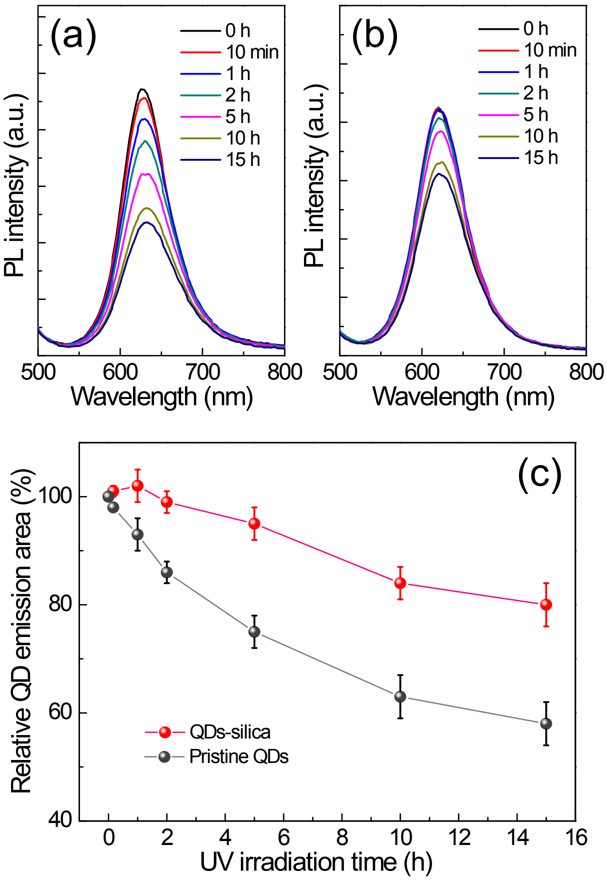

Fig. S6 PL spectra evolutions of (a) pristine QDs and (b) QDs-silica with UV (365 nm) irradiation exposure time. (b) Changes in relative QD emission area of pristine QDs and QDs-silica calculated from Fig. S4(a,b).

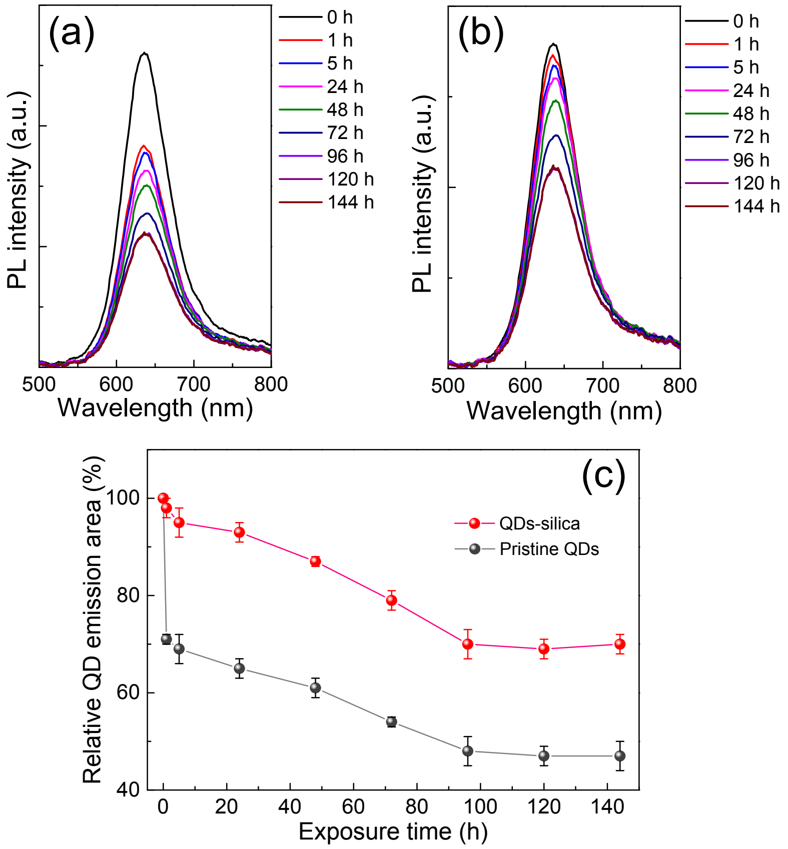

Fig. S7 PL spectra evolutions of (a) pristine QDs and (b) QDs-silica under 85°C and 85% RH for an extended period of time. (b) Changes in relative QD emission area of pristine QDs and QDs-silica calculated from Fig. S5(a,b).

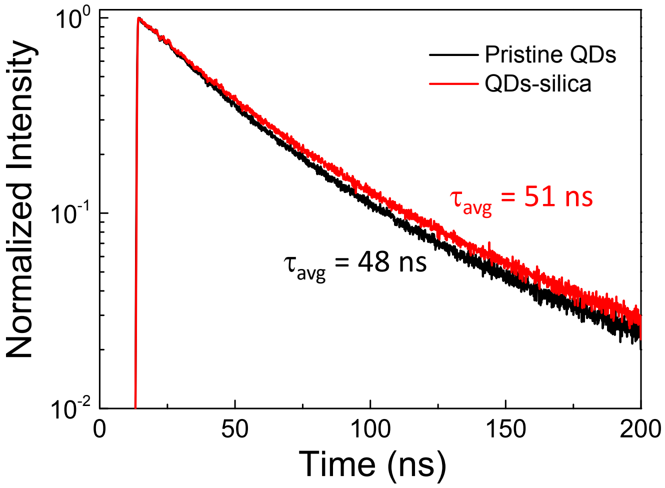

Fig. S8 PL decay curves and average lifetime (τ_{avg}) values of pristine QDs and QDs-silica powders. The samples were measured in the form of cured resin mixture on glass substrate. Here, τ_{avg} denotes the time when PL intensity corresponds to 1/e of the initial PL intensity.

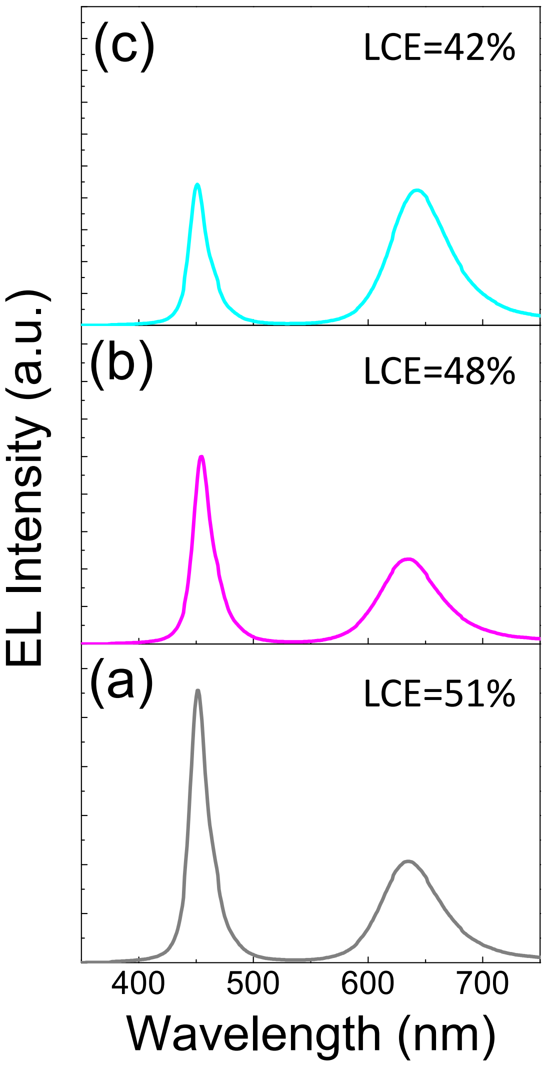

Fig. S9 Comparison of EL spectra and the resulting LCEs of on-chip-packaged QD-LEDs with different loading amounts of QDs-silica powder collected at an input current of 60 mA.; (a) 13, (b) 18, and (c) 25 mg.
